# Synthesis and characterization of iodovanadinite using PdI_2,_ an iodine source for the immobilisation of radioiodine†

**DOI:** 10.1039/d0ra04114a

**Published:** 2020-07-02

**Authors:** E. V. Johnstone, D. J. Bailey, S. Lawson, M. C. Stennett, C. L. Corkhill, M. Kim, J. Heo, D. Matsumura, N. C. Hyatt

**Affiliations:** University of Sheffield, Materials Science and Engineering Department Sheffield S1 3JD UK n.c.hyatt@sheffiled.ac.uk; Department of Materials Science and Engineering, Pohang University of Science and Technology (POSTECH) Pohang Gyeongbuk 790-784 South Korea; Division of Advanced Nuclear Engineering, Pohang University of Science and Technology (POSTECH) Pohang Gyeongbuk 790-784 South Korea; Materials Sciences Research Center, Japan Atomic Energy Agency 1-1-1 Koto Sayo Hyogo 679-5148 Japan

## Abstract

The synthesis of a palladium-containing iodovanadinite derivative, hypothetically “PdPb_9_(VO_4_)_6_I_2_”, was attempted using PdI_2_ as a source of iodine in searching for a novel waste form for radioiodine. Stoichiometric amounts of Pb_3_(VO_4_)_2_ and PdI_2_ were batched and reacted at elevated temperatures in sealed vessels. Batched material was also subjected to high-energy ball-milling (HEBM) in order to reduce reaction time and the potential for iodine volatilization during subsequent reaction at 200–500 °C. The resulting products were characterized using X-ray diffraction, scanning electron microscopy, energy-dispersive X-ray analysis, IR spectroscopy, thermal analysis and Pd K XANES. Results showed that PdI_2_ can function as a sacrificial iodine source for the formation of iodovanadinite, prototypically Pb_10_(VO_4_)_6_I_2_, however, the incorporation of Pd into this phase was not definitively observed. The sacrificial reaction mechanism involved the decomposition of PdI_2_ to Pd metal and nascent I_2_, with the latter incorporated into the iodovanadinite Pb_10_(VO_4_)_6_I_2_ phase. In comparison to processing using standard solid state reaction techniques, the use of HEBM prior to high temperature reaction generates a more homogeneous end-product with better iodine retention for this system. Overall, the key novelty and importance of this work is in demonstrating a method for direct immobilisation of undissolved PdI_2_ from nuclear fuel reprocessing, in a composite wasteform in which I-129 is immobilised within a durable iodovandinite ceramic, encapsulating Pd metal.

## Abstract 2

The synthesis and characterisation of a composite wasteform, comprising iodovanadinite Pb_10_(VO_4_)_6_I_2_ and Pd metal, is reported, for immobilisation of radioiodine PdI_2_; the formation of Pd incorporated iodovanadinite “PdPb_9_(VO_4_)_6_I_2_” was not observed.

## Graphical abstract

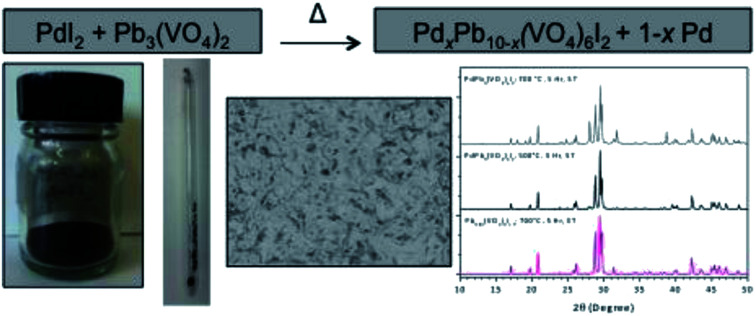

## Introduction

Iodine-129 is a by-product produced from the fission of actinide fuels. Due to its unique chemical behaviour and long half-life (15.7 × 10^6^ years), ^129^I is a challenge for radioactive waste disposal where containment is essential.^1,2^ In the past, sources of intentional and accidental radioiodine releases into the biosphere have included a variety of nuclear activities, such as reprocessing,^3^ reactor or medical isotope generator accidents,^4,5^ and weapons testing.^6^

Silver (^109^Ag) and a distribution of palladium isotopes (^105^Pd, ^106^Pd, ^108^Pd, ^110^Pd) including one long-lived radiopalladium isotope, *i.e.*, ^107^Pd (*t*_1/2_ = 6.5 × 10^6^ years),^7^ are fission products formed congruently with radioiodine in the fuel during nuclear energy production. These elements have been found within undissolved solids (UDS) in nuclear fuel reprocessing, alloying with other fission product metals (Mo, Tc, Ru, Rh),^8^ and as colloidal solids of AgI, PdI_2_ and (Ag,Pd)I.^9–11^ Whereas the direct disposal of AgI and PdI_2_ metal salts has been suggested,^1^ their immobilisation within a stable tailored glass and/or ceramic waste form has also been proposed. Iodovanadinite, prototypically Pb_10_(VO_4_)_6_I_2_, is one potential ceramic matrix for immobilisation of radioiodine,^12–19^ due to its chemical durability,^20–22^ radiation tolerance,^23^ and its ability to effectively accommodate the sterically demanding iodide anion within the one dimensional tunnels of the apatite crystal structure.^13,17^

Whereas a majority of the reported work on Pb_10_(VO_4_)_6_I_2_ has focused on synthesis of this material,^15–19,23^ few studies have considered the use of different iodine sources other than PbI_2_. It was previously reported that it was possible to incorporate AgI into a silver-containing, iodine-deficient iodovanadinite variant, AgPb_9_(VO_4_)_6_I;^24^ however, recent efforts have shown that the actual phase assemblage primarily comprised AgI and β-Pb_3_(VO_4_)_2_.^25^

From our extensive review of the relevant literature, no studies have been aimed at incorporating PdI_2_ into a secondary host ceramic or glass waste form. The motivation for this approach is to utilise the iodine bearing compounds that could be extracted from the residue of fuel dissolution, without conversion to a more amenable reagent, such as PbI_2_. Therefore, it was of interest to investigate the use of PdI_2_ as an iodine-bearing reagent for the synthesis of Pd-containing iodovanadinite phase.

The aim of this study was to synthesize a ceramic matrix for the sequestration and disposal of radioiodine, in the initial form of PdI_2_, relevant to speciation in the residue derived from reprocessing of spent nuclear fuel. Herein, we report the reaction of β-Pb_3_(VO_4_)_2_ and PdI_2_, with the objective of forming the iodovanadinite derivative “PdPb_9_(VO_4_)_6_I_2_”, by high temperature solid state reactions and high-energy ball-milling (HEBM) methods. The reaction products were studied using X-ray diffraction, electron microscopy with coupled energy dispersive X-ray analysis, thermal analysis, and infra-red and X-ray spectroscopy techniques, to determine the phase assemblage and compositions.

## Experimental

### Materials

β-Pb_3_(VO_4_)_2_ was prepared from the solid–state reaction of PbO and V_2_O_5_ in a 3 : 1 stoichiometry at 800 °C for 12 h in air. Pb_9.85_(VO_4_)_6_I_1.7_ was synthesized as reported in the literature.^23,25^ PdI_2_ and other chemicals were purchased from Sigma-Aldrich and used as received.

### Synthesis

Stoichiometric amounts of β-Pb_3_(VO_4_)_2_ and PdI_2_ (3 : 1) were batched for a ∼2 g sample of “PdPb_9_(VO_4_)_6_I_2_”. The batched powders were pulverized for 5 minutes in a mini-ball mill (Fritsch Pulverisette 23) equipped with a ∼10 mL zirconium oxide pot and ∼5 g of spherical grinding media, using ethanol as a carrier fluid. The resulting slurry was dried and passed through a coarse sieve mesh. The resulting black-grey powder was pressed into ∼250 mg disk pellets, and the pellets were reacted in evacuated sealed quartz tubes in an alumina tube furnace at 500 and 700 °C for 5 h.

### High-energy ball-milling (HEBM) synthesis

Stoichiometric amounts of β-Pb_3_(VO_4_)_2_ and PdI_2_ (3 : 1) were batched for a ∼5 g sample of “PdPb_9_(VO_4_)_6_I_2_”. The batched powders were pulverized by HEBM (500 rpm with cycles of 10 min on and 5 min off, followed by a reversal in direction) with a planetary ball mill (Fritsch Pulverisette 7 Premium Line) in a ∼30 mL silicon nitride pot with 23 g of spherical grinding media, using ethanol as a carrier fluid. The batch was subjected to HEBM for a total of 30 h, the time after which no further amorphisation of the product was observed, and samples were analysed after 1, 2.5, 5, 10, and 20 h periods. After 30 h, the resulting material was dried and passed through a coarse sieve mesh followed by a 200 μm sieve mesh. The resulting black-grey powder was pressed into ∼250 mg disk pellets and the pellets were reacted in evacuated sealed quartz tubes at 200, 300, 400, and 500 °C for 1 h. Herein, we use the formula “PdPb_9_(VO_4_)_6_I_2_” to refer to the targeted phase of the synthesis, although, as evidenced and discussed, this phase was not isolated and the actual phase assemblage comprises several other components.

### Characterization techniques

Powder X-ray diffraction (PXRD) was performed on a Bruker D2 Phaser system operating with Ni filtered Cu Kα radiation and a position sensitive detector. Samples were pulverized with an agate mortar and pestle, and dispersed on a low-background silicon holder. Measurements were made from 10° < 2*θ* < 50° with a step size of 0.02 increments at scan rate of 1.0 min^−1^. Phase analysis was completed using Diffrac.Suite Eva V.3 (Bruker) and Rietveld refinements were performed using Topas V.4.2 (Bruker). Scanning electron microscopy (SEM) and Energy-dispersive X-ray spectroscopy (EDX) measurements were performed at an accelerating voltage of 15 keV with backscattered electron (BSE) detection using a Hitachi TM3030 SEM equipped with a Quantax 70 EDX system. Pieces of reacted pellets were cold-mounted in an epoxy resin and polished to 1 μm using SiC grit paper and diamond polishing paste with a polishing cloth, and carbon coated prior to analysis. Specimens were prepared for transmission electron microscopy (TEM) characterisation by gently grinding synthesised powders in isopropanol and pipetting the suspended powders onto Cu grids lined with carbon holey film (Gatan). TEM was performed using a JEOL 2010F field emission gun TEM and a charge coupled device camera operating at 200 keV. EDX analysis was performed using an Oxford Instruments EDX detector and the Oxford ISIS software package. Thermogravimetric (TG) and differential thermal analysis (DTA) measurements (Netzsch Jupiter STA 449F3) of powdered samples were performed under flowing Ar(g) in an alumina crucible with a scan rate of 10 °C min^−1^ from 50 to 800 °C. Differential scanning calorimetry (DSC)-TG measurements were performed on the same setup from 30 to 600 °C with a scan rate of 10 °C min^−1^. IR data was collected (Perkin Elmer Frontier MIN/NIR) on samples pressed as KBr pellets in the region from 4000 to 400 cm^−1^ at 298 K. Pd K X-ray Absorption Near Edge Structure (XANES) data were acquired in fluoresence mode at the bending magnet beamline BL14B1 of the SPring-8 synchrotron, Japan.^26^ A Si(311) double crystal monochromator was used, and the emitted fluorescence was collected using a 36-element solid state detector. Data were processed and analysed using the ATHENA software package.^27^

## Results

### Synthesis and characterization of “PdPb_9_(VO_4_)_6_I_2_”

**Fig. 1:**
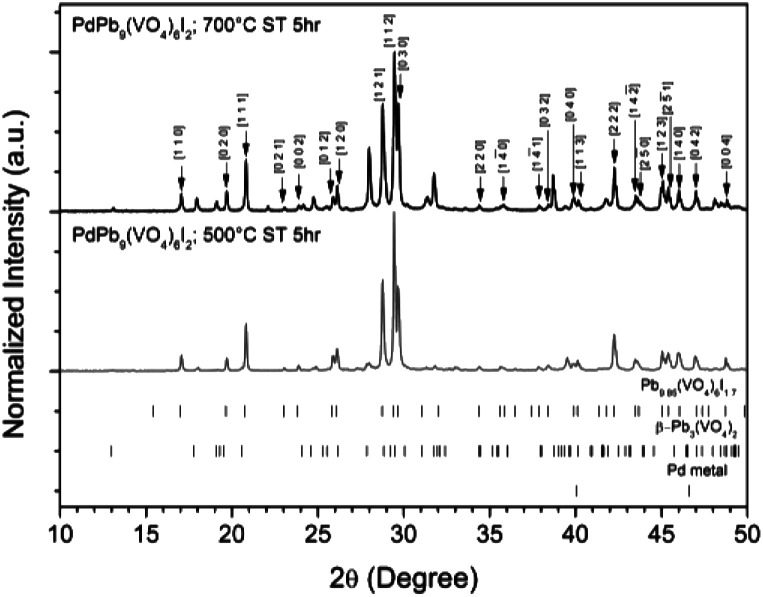
PXRD patterns of “PdPb_9_(VO_4_)_6_I_2_” reacted at 500 and 700 °C in a sealed quartz tube, indexed according to the published structure of Pb_9.85_(VO_4_)_6_I_1.7_.^17^ Tick marks at the bottom show allowed reflections for Pb_9.85_(VO_4_)_6_I_1.7_ (ICSD 280065; upper ticks), β-Pb_3_(VO_4_)_2_ (ICSD 29360; middle ticks), and Pd metal (COD 9009820; bottom ticks).

Powders of β-Pb_3_(VO_4_)_2_ and PdI_2_ in a 3 : 1 ratio were batched for the intended stoichiometric target composition of “PdPb_9_(VO_4_)_6_I_2_”. The batched compounds were ball-milled, pelletised, and reacted at elevated temperatures in sealed quartz tubes under vacuum. The phase assemblage of the reacted products was determined by PXRD with quantitative phase analysis using the Rietveld method (Fig. 1). Several phases were identified in the PXRD patterns, including Pb_9.85_(VO_4_)_6_I_1.7_, β-Pb_3_(VO_4_)_2_, and Pd metal. The product reacted at 500 °C was composed primarily of Pb_9.85_(VO_4_)_6_I_1.7_ (63.6(4) wt%) while the other two phases were minor in comparison (β-Pb_3_(VO_4_)_2_ 31.2(2) wt%); (Pd (6.1(1) wt%)). In contrast, the product obtained at 700 °C had noticeably more β-Pb_3_(VO_4_)_2_ (64.2(4) wt%) and less Pb_9.85_(VO_4_)_6_I_1.7_ (32.1(2) wt%) and Pd (1.0(1) wt%) present, consistent with the thermal decomposition data of Redfern *et al.*^28^ Rietveld analysis of the Pb_9.85_(VO_4_)_6_I_1.7_ phase (Table 1) in the sample reacted at 500 °C yielded comparable unit cell parameters to those reported from both single-crystal and PXRD measurements, indicating no significant structural distortion or gross deviation in composition.

**Table 1:** Calculated cell lattice parameters determined by Rietveld refinement for “PdPb_9_(VO_4_)_6_I_2_” reacted *via* standard-solid state techniques or *via* HEBM followed by low temperature reaction. Literature values are from single-crystal measurements determined for Pb_9.85_(VO_4_)_6_I_1.7_ and PXRD measurements from Pb_9.84_(VO_4_)_6_I_1.68_ synthesized by HEBM and reaction at 300 °C for 1 hour

Crystal system	“PdPb_9_(VO_4_)_6_I_2_”, 500 °C, 5 h, ST	HEBM “PdPb_9_(VO_4_)_6_I_2_”, 500 °C, 1 h, ST	Pb_9.85_(VO_4_)_6_I_1.7_ (ref. 13)	Pb_10_(VO_4_)_6_I_2_ (ref. 17)
	Hexagonal, *P*6_3_/*m*	Hexagonal, *P*6_3_/*m*	Hexagonal, *P*6_3_/*m*	Hexagonal, *P*6_3_/*m*
*a* (Å)	10.4684(2)	10.4580(3)	10.422(5)	10.4429(3)
*c* (Å)	7.4897(2)	7.4902(3)	7.467(3)	7.4865(3)

**Fig. 2:**
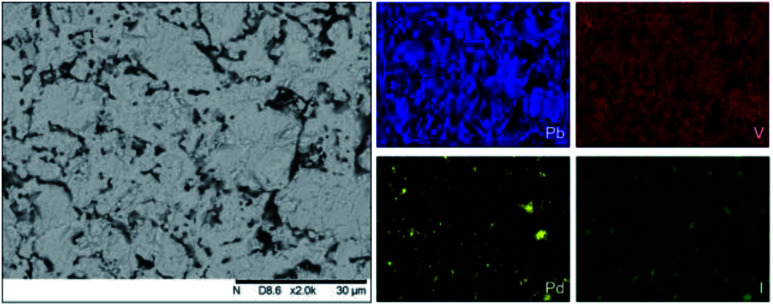
BSE SEM image at ×2000 magnification (scale bar length = 30 μm) and EDX map of Pb (blue), V (red), Pd (yellow), and I (green) present in “PdPb_9_(VO_4_)_6_I_2_” reacted at 500 °C for 5 h in a sealed quartz tube. EDX spectra are shown in Fig. S2.†

**Fig. 3:**
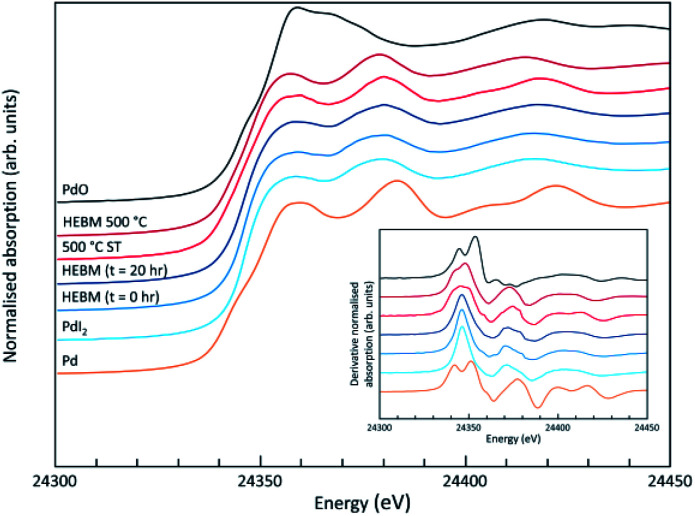
Pd K XANES data from “PdPb_9_(VO_4_)_6_I_2_” produced by conventional solid state reaction (500 °C ST) or High Energy Ball Milling (HEBM), with data shown for initial reaction mixture (HEBM 0 h), after 20 h (HEBM *t* = 20 h), and after subsequent reaction at 500 °C (HEBM 500 °C); plus reference compounds of Pd, PdI_2_ and PdO.

Analysis of the microstructure and phase assemblage for each sample was performed using SEM-EDX. For each sample, multiple phases were observed throughout the cross-sectioned area as shown in Fig. 2 and S1† for the 500 and 700 °C samples, respectively. In both samples a major phase was identified, composed of larger grains, forming the bulk network of the ceramic, while a minor phase, distributed as smaller particles within the grain boundaries, was also present. Element distribution maps of the area of interest identified the bulk phase as a composition of Pb, V, and I, whereas the small inclusions were determined to be two different phases: isolated Pd or Pd co-located with I. These phases would correspond to Pb_9.85_(VO_4_)_6_I_1.7_ and Pd metal, as determined by PXRD, and residual PdI_2_, respectively; the latter was not evident in PXRD data, likely due to complex powder pattern and consequent reflection overlap. The EDX semi-quantitative analysis of Pb, Pd, V, and I for the full-field view in Fig. 2 is shown in Table 2 (note: oxygen content was assumed to be stoichiometric, given the poor sensitivity of EDS towards oxygen determination). It is noted that the Pd concentration was lower and the Pb concentration higher than the calculated values for the target “PdPb_9_(VO_4_)_6_I_2_” phase, which is likely due to the overlap of the Pd Lα_1_ and Pb Mα_1_ X-ray emission lines used for quantification (at 2345.5 and 2838.6 eV, respectively), whereas the other element concentrations were within the standard margin of error. Further insight was provided by Pd K XANES data, shown in Fig. 3. The Pd K XANES of “PdPb_9_(VO_4_)_6_I_2_” reacted at 500 °C was found to be similar to that of Pd metal, although the profile of the post edge features also suggested some similarity to those of PdI_2_. A linear combination fit of the data of “PdPb_9_(VO_4_)_6_I_2_” reacted at 500 °C, using data of the reference compounds, afforded a satisfactory fit with weightings of 0.56 Pd and 0.43 PdI_2_ (*R* factor = 0.012; fractions constrained to sum to 1.0), consistent with XRD and SEM-EDX data; see Fig. S3.†

**Table 2:** Elemental analysis determined by EDX of “PdPb_9_(VO_4_)_6_I_2_” treated at 500 °C for 5 h under vacuum in a sealed vessel

Element	Calc. atomic%	500 °C 5 h ST, measured atomic%
Pb	50.0	53 ± 2
Pd	5.6	1.8 ± 0.1
V	33.3	34.4 ± 0.3
I	11.1	11.2 ± 0.3

In order to determine whether any Pd was incorporated into the iodovanadinite phase, TEM equipped with qualitative EDX was utilized to analyse several different single-grains of the material synthesized at 500 °C. The results show a material that definitively contained Pb, V, O, and I, presumably the targeted iodovanadinite host phase, as shown in Fig. S4;† however, the Pd Lα_1_ energy peak at 2838.6 eV was not readily discernible above the background. From these data, there is no evidence for significant Pd incorporation within the apatite phase.

**Fig. 4:**
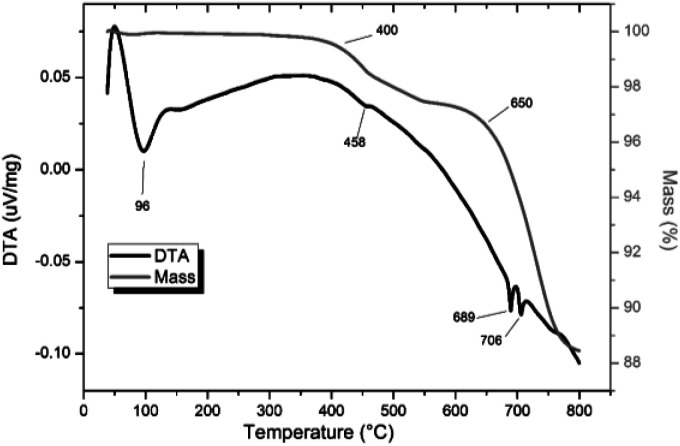
TGA-DTA analysis of “PdPb_9_(VO_4_)_6_I_2_” reacted in a sealed vessel under vacuum at 500 °C for 5 h.

TG-DTA analysis (Fig. 4) of “PdPb_9_(VO_4_)_6_I_2_”, reacted at 500 °C for 5 h in a sealed tube, showed that the phase assemblage was thermally stable up to ∼350 °C. Above this temperature, a small weight loss (∼2.5%) in the TG curve was observed, most likely due to the decomposition of PdI_2_ starting at ∼350 °C. A second, more significant drop in the TG curve was observed from ∼650 to 780 °C, corresponding to a mass loss of about 8%, attributed to the decomposition of the Pb_9.85_(VO_4_)_6_I_1.7_ phase. Previous studies on the thermal behaviour of Pb_9.85_(VO_4_)_6_I_2_ determined the compound to be stable up to 526 °C under a flowing Ar(g) atmosphere.^14^

### HEBM synthesis and characterization

**Fig. 5:**
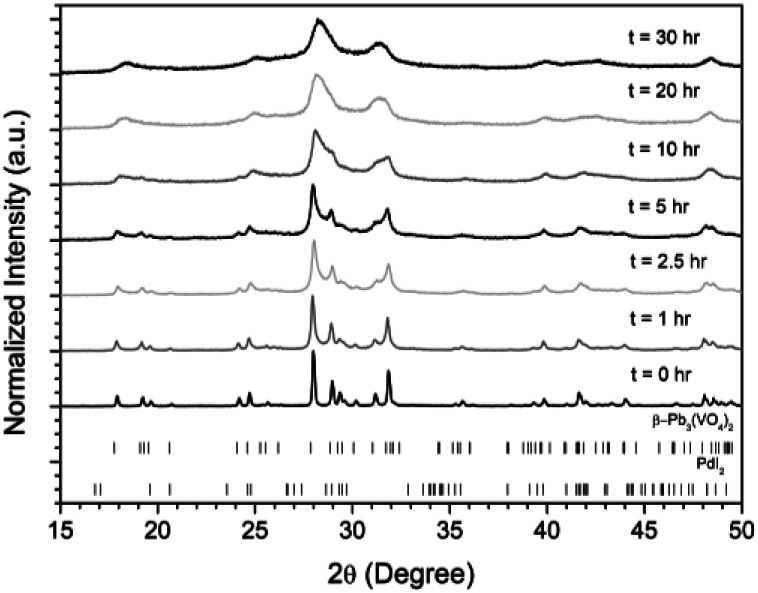
PXRD patterns of batched β-Pb_3_(VO_4_)_2_ and PdI_2_ subjected to HEBM after *t* = 0, 1, 2.5, 5, 10, 20, and 30 h. Respective tick marks represent allowed reflections for β-Pb_3_(VO_4_)_2_ (ICSD 1008164; top ticks) and PdI_2_ (ICSD 1539344; bottom ticks).

**Fig. 6:**
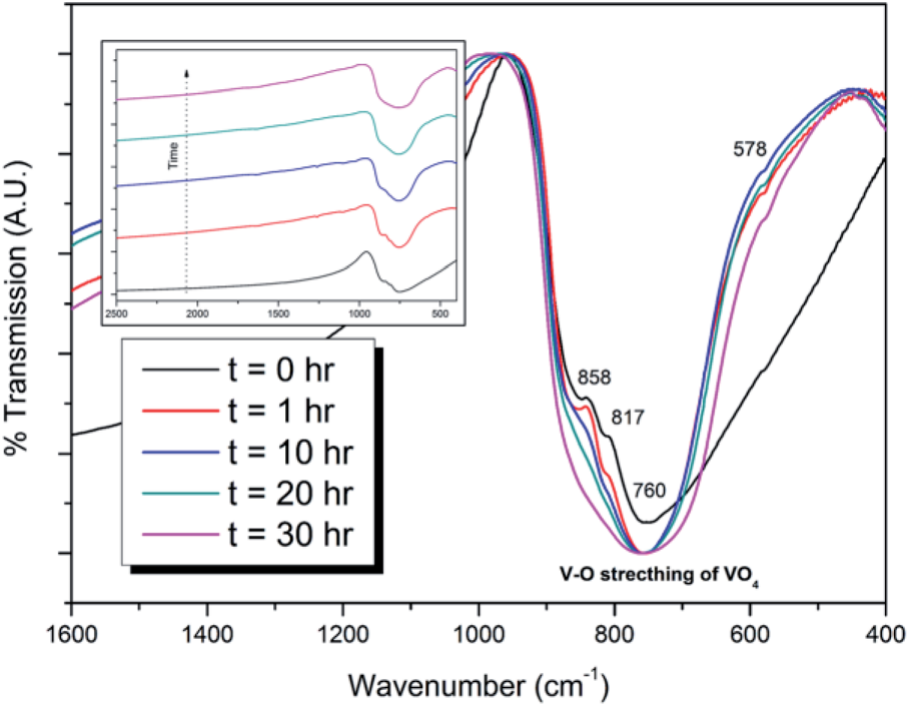
IR spectra of batched β-Pb_3_(VO_4_)_2_ and PdI_2_ subjected to HEBM after *t* = 0, 1, 10, 20, and 30 h. Inset: stacked IR spectra showing amorphisation of V–O stretching in the VO_4_ unit as a function of HEBM time.

Powders of β-Pb_3_(VO_4_)_2_ and PdI_2_ in a 3 : 1 ratio were batched to yield the target composition of “PdPb_9_(VO_4_)_6_I_2_”. The resulting material was subjected to HEBM for a total of 30 h, producing a fine, dark grey to black powder. PXRD analysis (Fig. 5) of the powder after various times during HEBM showed the gradual amorphisation of the starting materials, predominantly of the characteristic reflections of β-Pb_3_(VO_4_)_2_ between 2*θ* = 27 to 33°. Amorphisation in HEBM arises from the accumulation of point defects induced by shear and plastic deformation induced by high energy impacts, which result in a solid state crystalline to amorphous phase transition, with increasing milling time.^29^ In particular, a clear change in the intensity and full width at half maximum (FWHM) of the β-Pb_3_(VO_4_)_2_ reflections was observed between the *t* = 5 and 30 h samples (Fig. S5†). After 30 h of HEBM, the PXRD pattern of the powder was characterized by low intensity, broad reflections typical of a nano-crystalline material, together with significant diffuse scattering attributed to an amorphous component; reflection maxima were observed at 2*θ* = 18.3, 25.0, 28.3, 31.4, 39.9, and 48.4°, with the most intense reflections at 2*θ* = 28.3 and 31.4°, consistent with the expected major reflections of β-Pb_3_(VO_4_)_2_. In comparison with the literature, the amorphised iodovanadinite assemblage, produced by HEBM of PbO, V_2_O_5_ and PbI_2_ for a minimum duration of 10 h, was characterized by a single peak occurring at ∼2*θ* = 28°.^30,31^ This difference in behaviour could plausibly be due to different milling conditions and the use of β-Pb_3_(VO_4_)_2_ as a starting material in this study, compared to binary metal oxides/iodide in the previous studies. It is noted that Suetsugu *et*. *al*. suspected the presence of Pb_3_(VO_4_)_2_ in their material, although the phase was not detectable by XRD.^31^ Consistent with PXRD data, IR analysis of the HEBM powders (Fig. 6) also indicated that extensive amorphisation of the starting material had occurred. This was observed for the V–O stretching in the VO_4_ unit (∼950 to 500 cm^−1^) in β-Pb_3_(VO_4_)_2_, and more specifically for the three characteristic bands at ∼858, 817, and 760 cm^−1^,^32^ which are prominent in the initially batched material at *t* = 0 h and after *t* = 1 h, but not the other samples. Progressive broadening of the band centred at 760 cm^−1^ was also observed with increasing durations of HEBM for the *t* = 1 to *t* = 30 h samples.

**Fig. 7:**
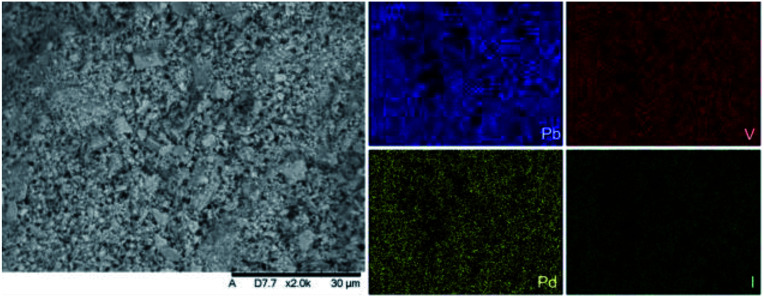
SEM-BSE image (×2000 magnification) and accompanying EDX elemental distribution plots for Pb, V, Pd, and I of batched powders of β-Pb_3_(VO_4_)_2_ and PdI_2_ subjected to HEBM after 30 h. Scale bar shown at bottom of SEM image represents 30 μm. EDX spectra are shown in Fig. S2.†

**Fig. 8:**
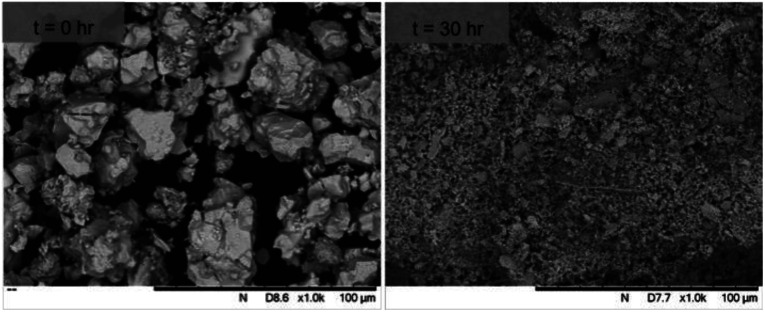
SEM images (×1000 magnification) showing particle size and morphology of initially batched powders (*t* = 0) and after HEBM *t* = 30 h. Scale bars shown at the bottom right of each image represent 100 μm.

**Fig. 9:**
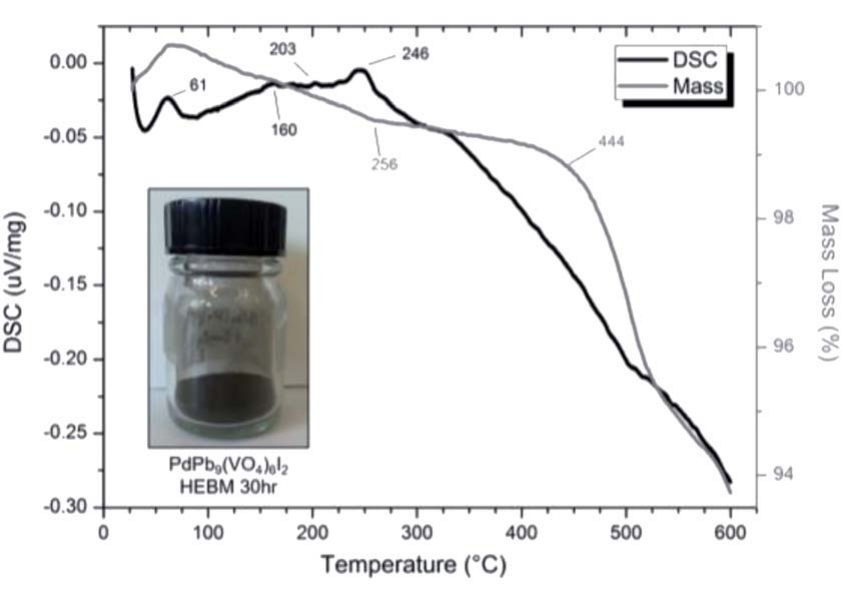
DSC (black line) and TGA (grey line) curves from 30 to 600 °C under flowing Ar(g) of “PdPb_9_(VO_4_)_6_I_2_” batched powders after 30 h HEBM. Insert: resulting powder after 30 h HEBM.

SEM imaging (Fig. 7 and 8) was used to characterise the particle size of the powder subjected to HEBM for *t* = 30 h. Particles sizes were distributed with some larger aggregates from 100 to 300 μm, but with a primary particle size ≤ 1 μm, which constituted the majority of the product. Elemental analysis as determined by EDX of the powder showed an even distribution of Pb, Pd, V, and I throughout the powder (Fig. 7). Pd K XANES data of the powder produced by HEBM for *t* = 30 h are presented in Fig. 3 and are a close match to those of the PdI_2_ reference compound. A linear combination fit of the data of, using data of the reference compounds, afforded a satisfactory fit with weightings of 0.18 Pd and 0.82 PdI_2_ (*R* factor = 0.018; fractions constrained to sum to 1.0); see Fig. S3.† Thus, the PdI_2_ reagent remains essentially unreacted during the HEBM process, although a minor fraction may decompose to Pd metal. TG-DTA characterisation of the HEBM material produced after 30 h (Fig. 9), showed two notable endothermic signals at 61 and 246 °C as well as two less intense signals at 160 and 203 °C. The peak located at 61 °C can be assigned to adsorbed water, whereas the peak at 246 °C is attributed to the formation of an iodovanadinite-like phase. In comparison to the DSC data reported by Lu *et al.* for Pb_10_(VO_4_)_6_I_2_ after 10 h of HEBM, a single peak associated with formation of the iodovanadinite phase was identified at ∼228 °C, which after longer HEBM treatment was shifted to ∼209 °C.^30^ The higher temperature of crystallisation/formation here is likely due to the residual crystallinity of the β-Pb_3_(VO_4_)_2_ reagent and unreacted PdI_2_ mixture, *versus* the effectively amorphous precursor produced from binary oxide reagents. Still, this formation temperature is notably lower than that of the material not subjected to HEBM, including that reported for Pb_9.85_(VO_4_)_6_I_1.7_.^13^ Significant mass loss was not observed in the TGA plot until ∼444 °C, which can be attributed to iodine volatilization.

**Fig. 10:**
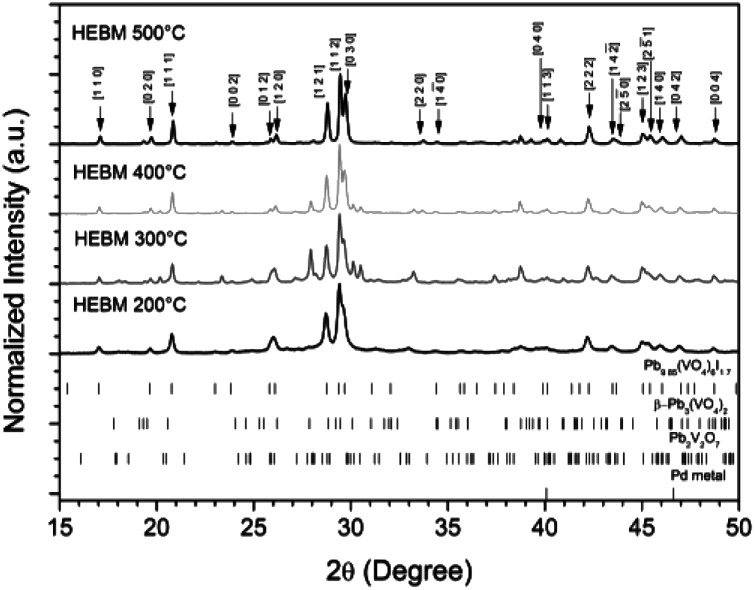
PXRD patterns of HEBM “PdPb_9_(VO_4_)_6_I_2_” after reaction at 200, 300, 400, and 500 °C for 1 h in sealed tubes. Markers on graph indicate peaks assigned to the Pb_9.85_(VO_4_)_6_I_1.7_ (labelled (*hkl*) Miller indices) phase. Respective tick marks represent allowed reflections for Pb_9.85_(VO_4_)_6_I_1.7_ (ICSD 280065), β-Pb_3_(VO_4_)_2_ (ICSD 1008164), Pb_2_V_2_O_7_ (ICSD 21020), and Pd metal (ICSD 52251).

The powder material produced by HEBM for 30 h was pelletised, sealed under vacuum in quartz tubes, and reacted at elevated temperatures between 200 to 500 °C for 1 h to determine the effect of heat treatment on phase assemblage and iodine retention/volatilisation. Each reaction yielded dark grey to black pellets that produced dark grey to black powders upon grinding. PXRD data from the heat treated materials is shown in Fig. 10. The iodovanadinite phase is formed at reaction temperatures as low as 200 °C after 1 h, 300 °C lower than that reported for the standard synthesis of Pb_9.85_(VO_4_)_6_I_1.7_.^13^ The crystallinity of the material, including the iodovanadinite phase, increased with increasing reaction temperatures. At intermediate temperatures, *i.e.*, 300–400 °C, secondary phases, such as β-Pb_3_(VO_4_)_2_ and Pb_2_V_2_O_7_, were detected, although they were absent in the material synthesized at 500 °C. Rietveld analysis of the iodovanadinite phase produced by HEBM for 30 h and heat treatment at 500 °C, afforded similar unit cell parameters to those obtained for the standard solid state synthesis at 500 °C and those reported in the literature (Table 1). Quantitative phase analysis of the material synthesised at 500 °C for 1 h afforded: 74.7(5) wt% Pb_9.85_(VO_4_)_6_I_1.7_, 11.0(1) wt% β-Pb_3_(VO_4_)_2_, 13.8(1) wt% Pd.

**Fig. 11:**
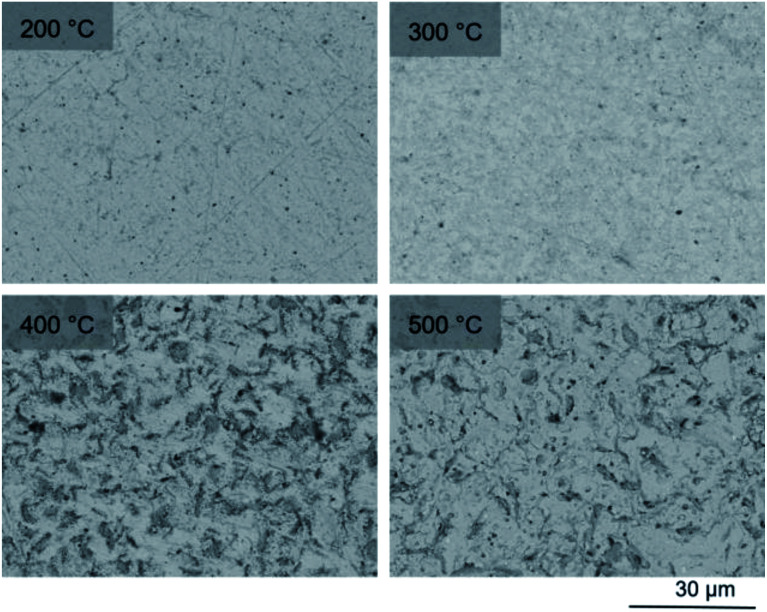
SEM images (×2000 magnification) in BSE mode of sectioned “PdPb_9_(VO_4_)_6_I_2_” pellets reacted in sealed tubes at 200, 300, 400, and 500 °C for 1 h. Scale bar presented at the bottom right-hand corner represents 30 μm.

**Fig. 12:**
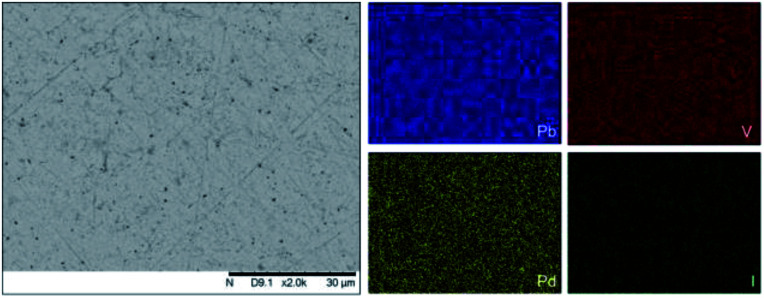
BSE SEM image at ×2000 magnification (scale bar length = 30 μm) and EDX map of Pb (blue), V (red), Pd (yellow), and I (green) present in “PdPb_9_(VO_4_)_6_I_2_” HEBM reacted at 200 °C for 1 h in a sealed quartz tube. EDX spectra are shown in Fig. S2.†

**Fig. 13:**
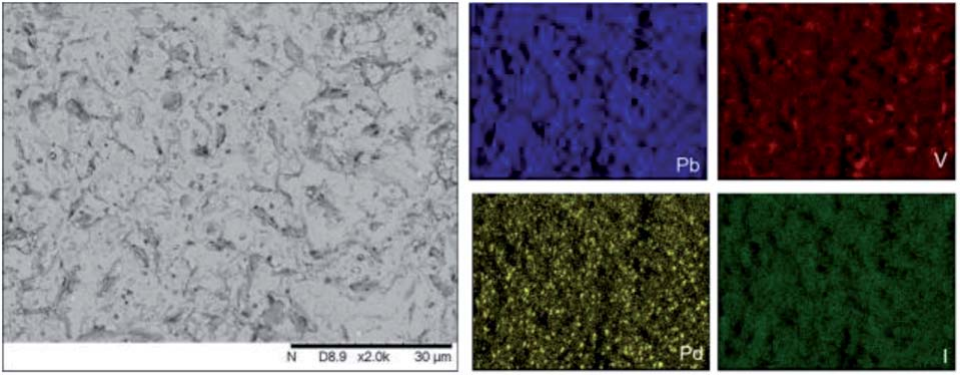
BSE SEM image at ×2000 magnification (scale bar length = 30 μm) and EDX map of Pb (blue), V (red), Pd (yellow), and I (green) present in “PdPb_9_(VO_4_)_6_I_2_” HEBM reacted at 500 °C for 1 h in a sealed quartz tube. EDX spectra are shown in Fig. S2.†

To observe the microstructure and phase assemblage of the products from HEBM for 30 h and heat treatment, SEM analyses were performed on sectioned pellets (Fig. 11). For the sample prepared at 200 °C, what appears to be a single-phase material was observed; the corresponding elemental distribution map (Fig. 12) was consistent with this observation and Pb, V, Pd, and I were shown evenly dispersed throughout the matrix. The measured elemental composition of the field of view shown in Fig. 12, determined quantitatively by EDX (Table 3), was in agreement with the calculated stoichiometry of the target “PdPb_9_(VO_4_)_6_I_2_” phase. This phase is identified as the nano-crystalline Pb_9.85_(VO_4_)_6_I_1.7_ observed in the corresponding PXRD data. However, with increasing temperature the ingrowth of secondary phases was observed in the BSE images, and isolated Pd (lighter contrast) and V rich (darker contrast) phases were identified along with Pb_9.85_(VO_4_)_6_I_1.7_, consistent with interpretation of the PXRD data. This trend was also apparent in the element distribution plots for Pb, V, I, and Pd in the samples treated at 200 and 500 °C as shown in Fig. 12 and 13. Notably, Pd was found in these samples either as an isolated phase, presumably as the metal, or associated with the bulk iodovanadinite phase, however, it was not evident here whether Pd incorporation in the iodovanadinite had occurred due to resolution constraints of the SEM. Quantitative EDX analyses of the products, Table 3, determined a composition broadly consistent with that calculated for “PdPb_9_(VO_4_)_6_I_2_”, but with a notable decrease in Pd and I concentrations with increasing temperature, due to phase separation of these elements. Pd K XANES data of “PdPb_9_(VO_4_)_6_I_2_” produced by HEBM for *t* = 30 h and reaction at 500 °C are presented in Fig. 3. These data are similar to those of the sample prepared by solid state synthesis and a linear combination analysis, using data of the reference compounds, afforded a satisfactory fit with weightings of 0.30 Pd and 0.70 PdI_2_ (*R* = 0.12; fractions constrained to sum to 1.0), consistent with XRD and SEM-EDX data. However, it was evident from the analysis, Fig. S3,† that an additional component is required to fully describe the spectra, implying a third unknown Pd environment is present. TEM analysis on several single grains of HEBM “PdPb_9_(VO_4_)_6_I_2_” reacted at 500 °C for 1 h yielded similar results as the product produced from standard solid state reaction methods mentioned earlier. As shown in the TEM-EDX spectrum in Fig. S6,† only X-ray emission lines associated with Pb, V, O, and I were identified; thus, regardless of processing method, Pd incorporation into the iodovanadinite phase could not be substantiated under these conditions.

**Table 3:** Elemental analysis determined by EDX of “PdPb_9_(VO_4_)_6_I_2_” after HEBM for *t* = 30 h, and subsequent reaction at 200–500 °C for 1 h under vacuum in a sealed vessel

Element	Calc. atomic%	200 °C, measured atomic%	300 °C, measured atomic%	400 °C, measured atomic%	500 °C, measured atomic%
Pb	50.0	46 ± 2	50.0 ± 2	50 ± 2	50 ± 2
Pd	5.6	5.6 ± 0.1	4.2 ± 0.1	3.24 ± 0.09	2.66 ± 0.08
V	33.3	37.0 ± 0.4	36.0 ± 0.4	37.7 ± 0.3	38.6 ± 0.4
I	11.1	11.3 ± 0.3	10.2 ± 0.3	9.5 ± 0.2	8.3 ± 0.2

Thermal analyses of HEBM “PdPb_9_(VO_4_)_6_I_2_” reacted at 200 and 500 °C are shown in Fig. S7† and 14, respectively. Both samples were stable up until ∼540 °C, at which they exhibited a two-step decomposition. The first decomposition at ∼540 °C resulted in a ∼5% mass loss for both samples, while the second decomposition occurred at ∼670 °C for the HEBM “PdPb_9_(VO_4_)_6_I_2_” reacted at 200 °C, and ∼705 °C for HEBM “PdPb_9_(VO_4_)_6_I_2_” reacted at 500 °C, with mass losses ranging ∼9–10% attributed to iodine release for each. Both samples produced by HEBM exhibited improved thermal stability with higher initial and secondary decomposition temperatures than those produced by high temperature solid–state reaction, likely a result from the better reaction and product homogeneity. The reported values here are also consistent with those in the literature for the iodovanadinite phase, which report thermal stability up until ∼500 °C, after which, loss of iodine occurred.^14,30^

**Fig. 14:**
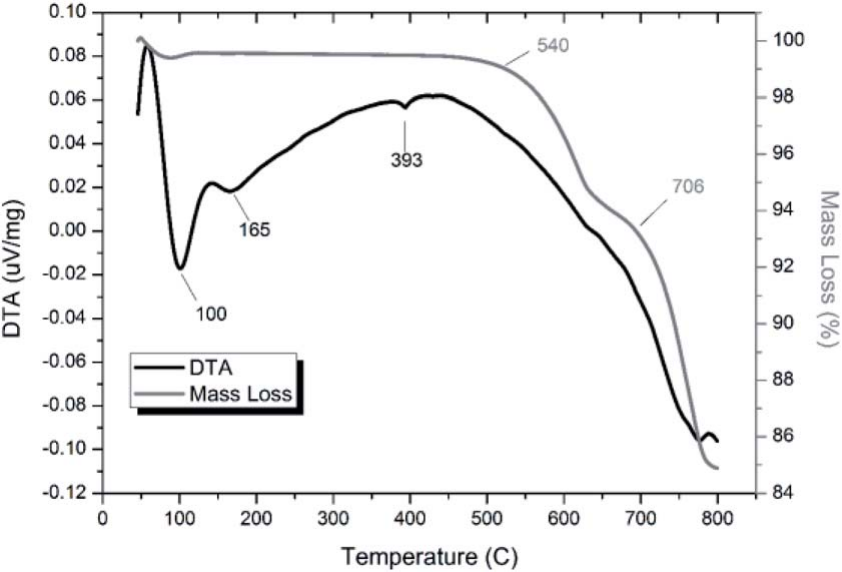
TGA-DTA analysis of “PdPb_9_(VO_4_)_6_I_2_” subjected to HEBM 30 h and reacted at 500 °C for 1 h in a sealed vessel under vacuum.

## Discussion

There have been numerous reports on M^2+^ substitutions into apatite related phases A_10_(BO_4_)_6_X_2_ (where A = Ca, Sr, Ba, Pb, Cd; B = P, V, As; X = OH, F, Cl, Br, I). In the case of Pb_9.85_(VO_4_)_6_I_1.7_, the Pd^2+^ (ionic radii in six-fold coordination, 0.86 Å) is considerably smaller in size than Pb^2+^ (1.19 Å) and lacks the active 6s^2^ electron lone pair of Pb^2+^, which could reasonably explain the absence of Pd within the iodovanadinite structure based on crystallographic/structural grounds. Indeed, no previous literature has reported Pd^2+^ substitution into any related A_10_(BO_4_)_6_X_2_ phase. We note that the doping of the significantly smaller Ta^5+^ (0.64 Å) for Ca^2+^ (1.00 Å) in chloroapatite, *i.e.* Ca_5_(PO_4_)_3_Cl, has been reported. The doped Ta^5+^ was found preferentially located at the Ca(i) site, where the smallest Ca–Ca distances are observed.^33^ In comparison with other possible cation substitutions, the ionic radius of Ag^+^ (1.15 Å) is more similar to that of Pb^2+^, which could explain the reported stability of AgPb_9_(VO_4_)_6_I and AgLnPb_8_(PO_4_)_6_X_2_ (X = F, Cl);^34,35^ however, as mentioned earlier, the purported iodine-deficient phase “AgPb_9_(VO_4_)_6_I” is actually a combination of AgI and Pb_3_(VO_4_)_2_, and Ag^+^ incorporation was not observed under similar circumstances as here.^25^ The 6-fold coordinate Cu^2+^ exhibits an ionic radius (0.73 Å) even smaller than that of Pd^2+^, but Cu^2+^ incorporation has been identified in natural samples of vanadinite, Pb_5_(VO_4_)_3_Cl, at concentrations ranging from 0.13 to 1.98%.^36^ Using Artificial Neural Network simulations of the apatite structure, Wang predicted that divalent A-site cations with ionic radii of ∼1.2 Å or larger (*e.g.*, Sr^2+^, Pb^2+^, Cd^2+^, Ba^2+^) in combination with pentavalent B-site cations (*e.g.*, V^5+^, As^5+^, Cr^5+^, Mo^5+^) would be structurally ideal for hosting iodine in the apatite channels.^37^ According to this analysis, it would not be expected that Pd^2+^ would be a suitable substitute for Pb^2+^ in iodovanadinite, which is consistent with the data presented herein. Solution syntheses of hydroxy- and fluoroapatites (HAP and FAP) with PdCl_2_(PhCN)_2_ for HAP/FAP-supported palladium catalysts have been reported;^38,39^ however, it was determined that Pd^2+^ substitution for Ca^2+^ did not occur, and rather Pd was surface bound by chemisorption.^36^ Similar behaviour has been observed for aqueous sorption experiments with Pd^2+^ and hydroxyapatite,^40^ which forms cationic surface complexes, such as PdOH^+^.

The reactivity and thermal behaviour the system was studied to better understand retention of iodine, a model for radioiodine handling in context of nuclear fuel reprocessing and disposal, where iodine volatilisation and release is problematic. It was of interest to study the use of an iodine-containing starting material, in particular one already occurring as a by-product in the nuclear fuel cycle (*i.e.*, PdI_2_) that could be directly incorporated, in comparison with that of the prototypical PbI_2_ reagent, into the iodovanadinite phase. However, one potential disadvantage of using PdI_2_ for these purposes is the lower decomposition temperature (∼350 °C) *versus* PbI_2_ (∼400 °C). Standard solid state reaction in a sealed system, using temperatures well above the decomposition temperature of PdI_2,_ yielded mixtures of iodovanadinite with Pd and PdI_2_. It is unlikely that the residual PdI_2_ was un-decomposed material, but rather *in situ* generated Pd metal reacted with liberated I_2_(g), not sequestered by the iodovanadinite phase, in the sealed system during cooling. Therefore, physical processing techniques, such as HEBM, which is capable of reducing reaction times and temperatures, were applied in order to compensate for the lower decomposition temperature. Interestingly, the phase assemblage and iodine incorporation was similar in materials produced by standard solid state reaction and HEBM at 500 °C, the thermal stability of the products was notably different. The HEBM material showed greater iodine retention at higher temperatures than the material synthesized by standard solid state reaction. Even the product of HEBM and low temperature reaction at 200 °C yielded a moderately high capacity to retain iodine, exhibiting bulk decomposition behaviour and iodine liberation (∼540 °C) much above the decomposition temperature of pure PdI_2_.

Furthermore, the methods and data presented here demonstrate the capability of impregnating a ceramic apatite phase (or decomposition product, *e.g.*, Pb_3_(VO_4_)_2_, or both) with a relatively uniform distribution of Pd metal particles ranging from several micron to submicron sizes *via* a PdI_2_ starting material. This approach could also find useful applications in synthesis of heterogeneous catalysts, which can be constructed by embedding these types of PGMs into a robust, inert substrate for conditions requiring high durability and reusability. Although the targeted host phase was not successfully synthesised in this study, the resulting ceramic phase assemblage achieved successful immobilisation of iodine and Pd, within a Pb_9.85_(VO_4_)_6_I_1.7_ matrix encapsulating metallic Pd particulates, which is a plausible composite wasteform given the known chemical durability and radiation tolerance of the apatite phase.^20–23^ Indeed, this opens the door to development of such tailored composite wasteforms as an alternative approach to iodine immobilisation.

## Conclusions

The synthesis of an iodovandinite derivative “PdPb_9_(VO_4_)_6_I_2_” were was investigated using PdI_2_ and β-Pb_3_(VO_4_)_2_ as reagents, for application as a ceramic immobilisation matrix for radioiodine originating from the nuclear fuel reprocessing. Using standard solid state reaction methods or HEBM followed by reaction at 200–500 °C in sealed vessels, the product was comprised of Pb_9.85_(VO_4_)_6_I_2_ accompanied by the presence of Pd metal. No detection of Pd in single-grain Pb_9.85_(VO_4_)_6_I_2_ material was observed, and it was instead found mostly as metallic particles or reformed PdI_2_ dispersed throughout the matrix. The thermal stability of the material showed slight improvements when compared to parent phase, even after short reaction times with HEBM. These results demonstrate a route for handling undissolved PdI_2_ waste, or a future wasteform in which PdI_2_ is generated. Likewise, the results also validate the feasibility of using a sacrificial metal iodide source for the synthesis of heterogeneous phase wasteforms, where iodine is integrated into a more stable matrix in the presence of a corrosion resistant metal, such as Pd, that could improve overall wasteform behaviour and durability. For the handling and disposal of radioiodine, especially occurring as undissolved metal halide precipitates, further investigations are necessary for developing better matrices and synthetic methods that could improve iodine retention.

## Conflicts of interest

There are no conflicts of interest to declare.

## Supplementary Material

RA-010-D0RA04114A-s001
