# Supplementary material for: Synthesis and characterization of iodovanadinite using PdI2, an iodine source for the immobilisation of radioiodine
Source: RSC Adv. 2020 Jul 2;10(42):25116–24. doi: 10.1039/d0ra04114a (PMC9055183; doi:10.1039/d0ra04114a)
Supplement: RA-010-D0RA04114A-s001 [file RA-010-D0RA04114A-s001.pdf]

Electronic Supplementary Material (ESI) for RSC Advances.
This journal is © The Royal Society of Chemistry 2020

Synthesis and characterization of lead iodovanadinite using PdI_2, an iodine source for the immobilisation of radioiodine

E. V. Johnstone,[a,b] D. J. Bailey,[a] S. Lawson,[a] M. C. Stennett,[a] C. L. Corkhill,[a]
M. Kim,[a,c,d] J. Heo,[c,d] D. Matsumura,[e] N. C. Hyatt[a*]

[a] University of Sheffield, Materials Science and Engineering Department, Sheffield, S1 3JD, UK.

[b] Current affiliation: Innovative Fuel Solutions, LLC., North Las Vegas, NV, USA.

[c] Department of Materials Science and Engineering, Pohang University of Science and Technology (POSTECH), Pohang, Gyeongbuk 790-784, South Korea.

[d] Division of Advanced Nuclear Engineering, Pohang University of Science and Technology (POSTECH), Pohang, Gyeongbuk 790-784, South Korea.

[e] Materials Sciences Research Center, Japan Atomic Energy Agency, 1-1-1 Koto, Sayo, Hyogo 679-5148, Japan.

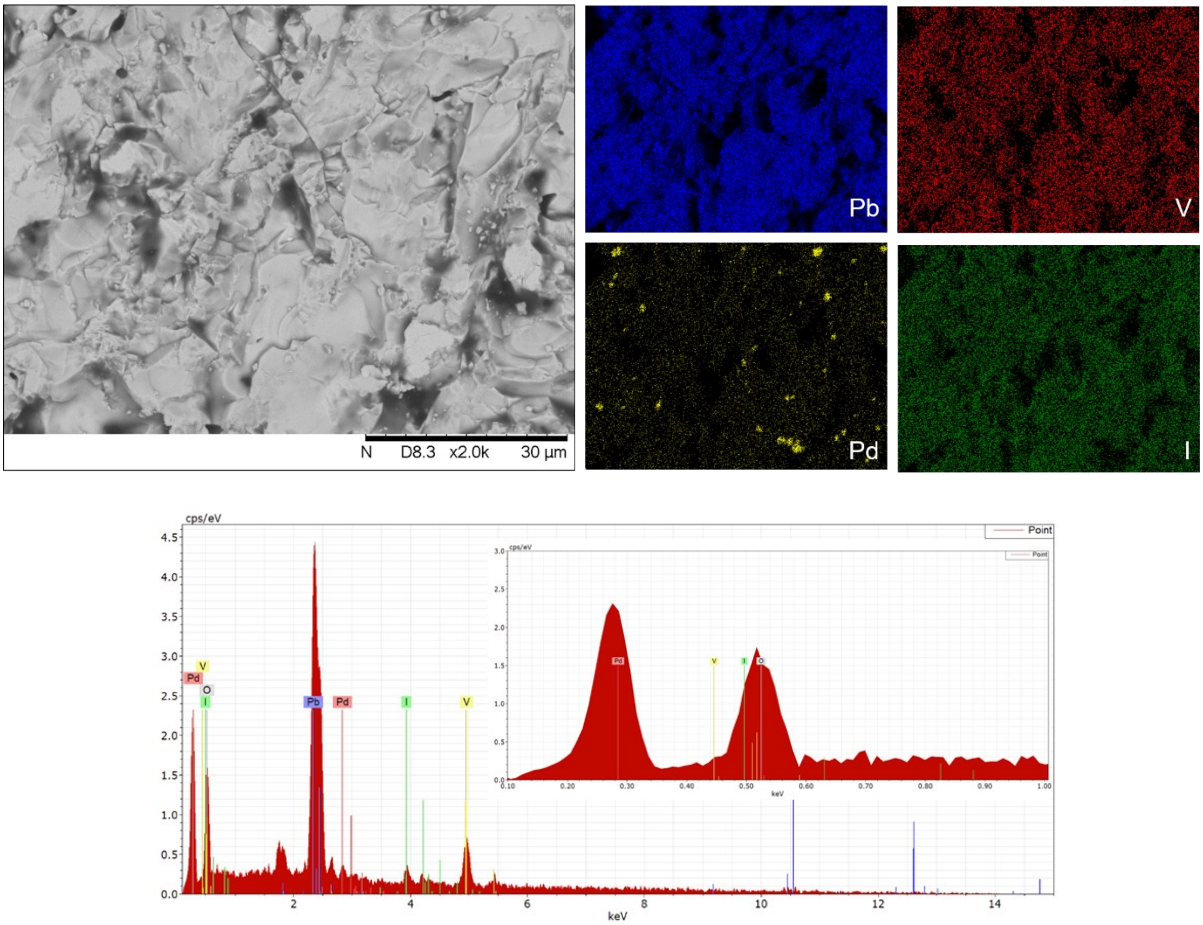

Figure S1. SEM-BSE image (x2000 magnification) and EDX elemental distribution plots for Pb, V, Pd, and I of a sectioned pellet of $PdPb_9(VO_4)_6I_2$ reacted at 700 °C for 5 hr. Scale bar shown at bottom of SEM image represents 30 µm. EDX spectrum corresponds to field of view in SEM-BSE and element distribution maps.

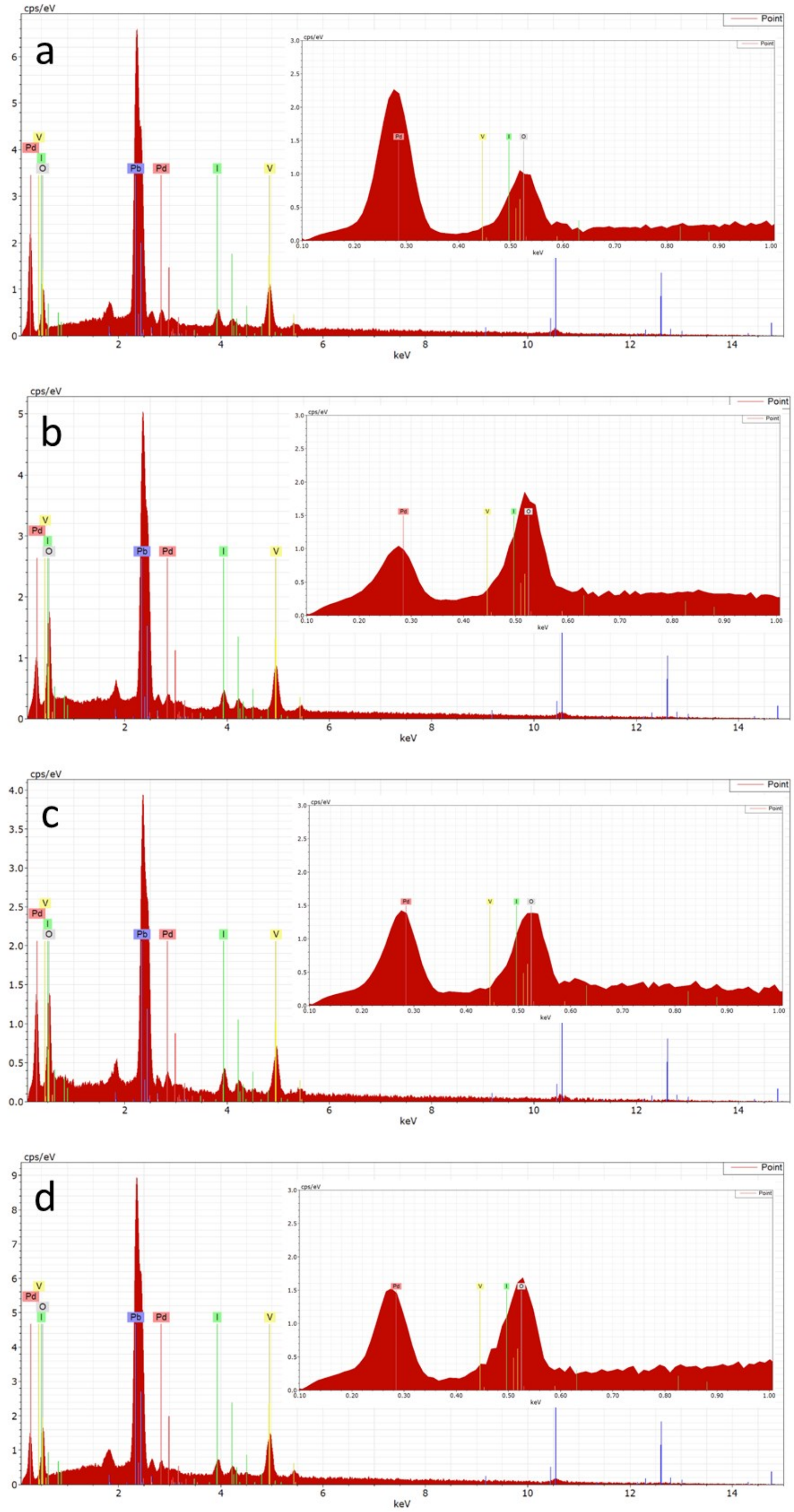

Figure S2. EDX spectra corresponding to field of view in SEM-BSE and element distribution maps in: a) Figure 2, b) Figure 7, c) Figure 12, d) Figure 13.

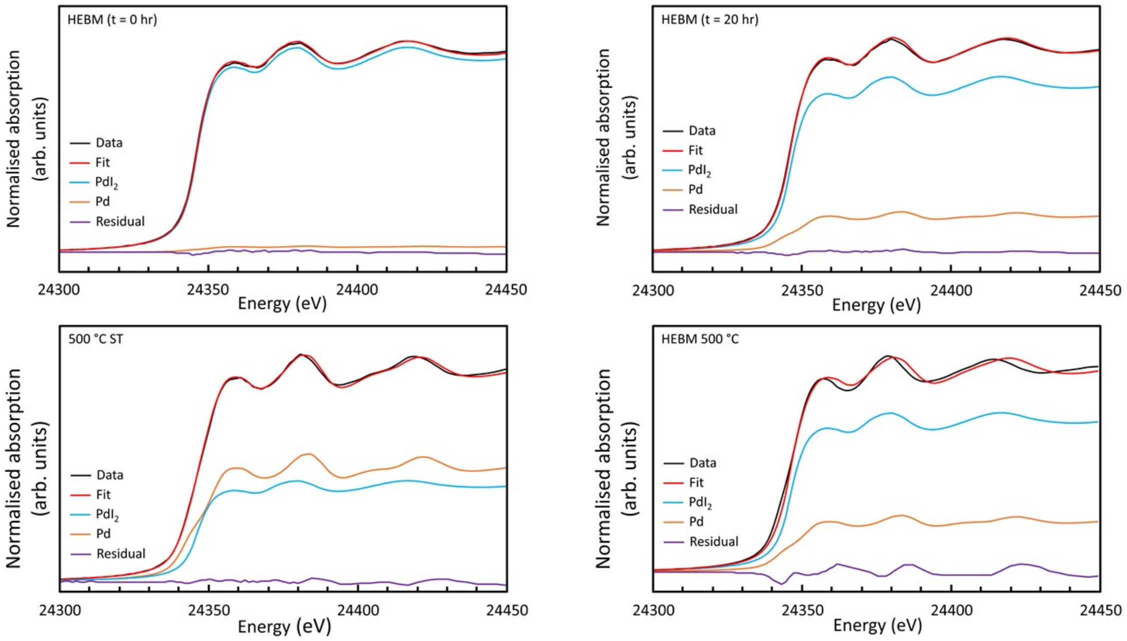

Figure S3. Linear combination fits to Pd L_3 XANES data from "$PdPb_9(VO_4)_6I_2$" produced by conventional solid state reaction (500°C ST) or High Energy Ball Milling (HEBM), with data shown for initial reaction mixture (HEBM 0hr), after 20 h (HEBM t = 20 hr), and after subsequent reaction at 500°C (HEBM 500°C); plus reference compounds of Pd, PdI_2 and PdO.

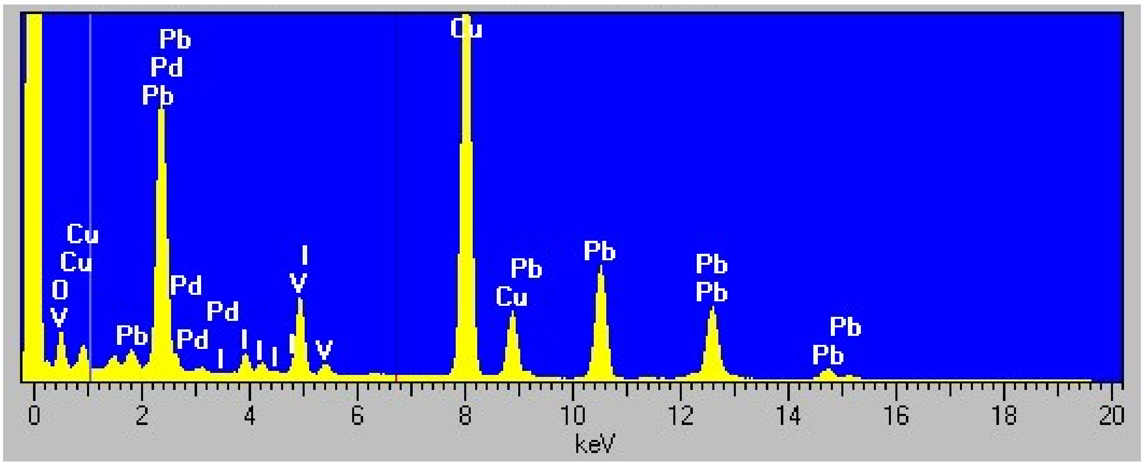

Figure S4. TEM-EDX spectrum of a single-grain of "$PdPb_9(VO_4)_6I_2$" reacted at 500 °C for 5 hr yielding peaks for Pb, Pd, V, O, and I..

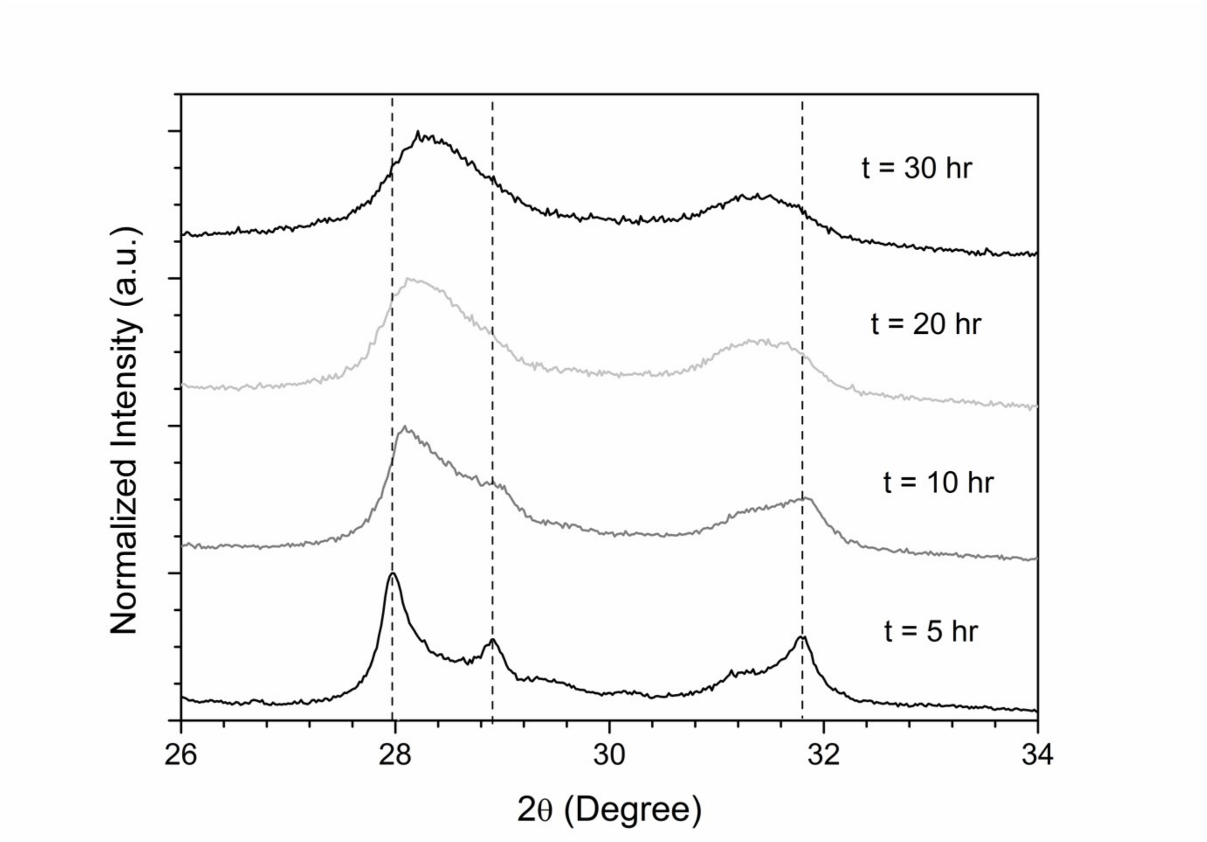

Figure S5. Expert from the PXRD patterns of batched β-$Pb_3(VO_4)_2$ and PdI_2 subjected to HEBM after t = 5, 10, 20, and 30 hr showing the amorphization and peak shift of the most intense reflections between 2θ = 27 to 33°.

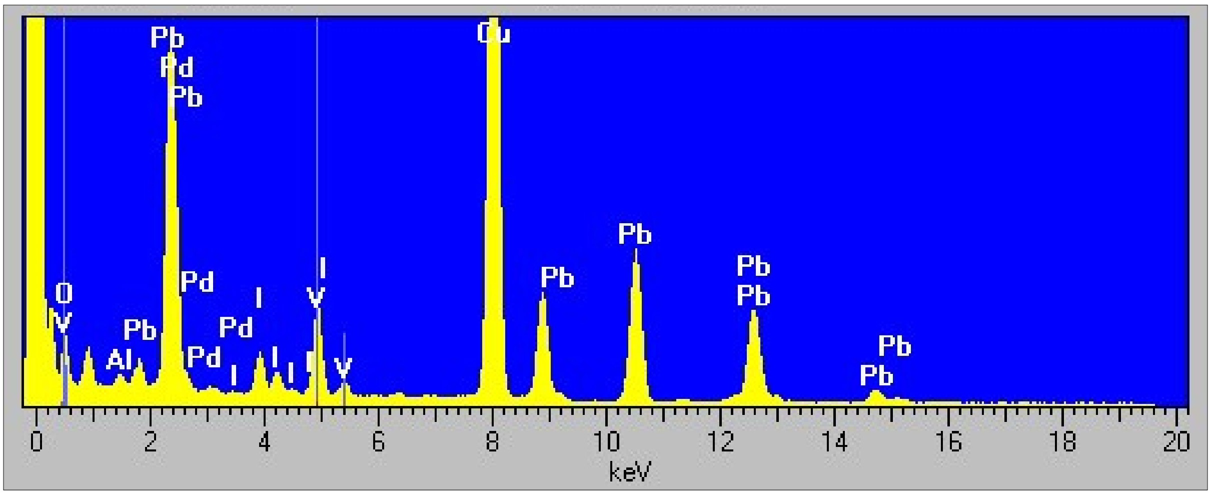

Figure S6. TEM-EDX spectrum of a single-grain of HEBM "$PdPb_9(VO_4)_6I_2$" reacted at 500 °C for 1 hr yielding peaks for Pb, Pd, V, O, and I.

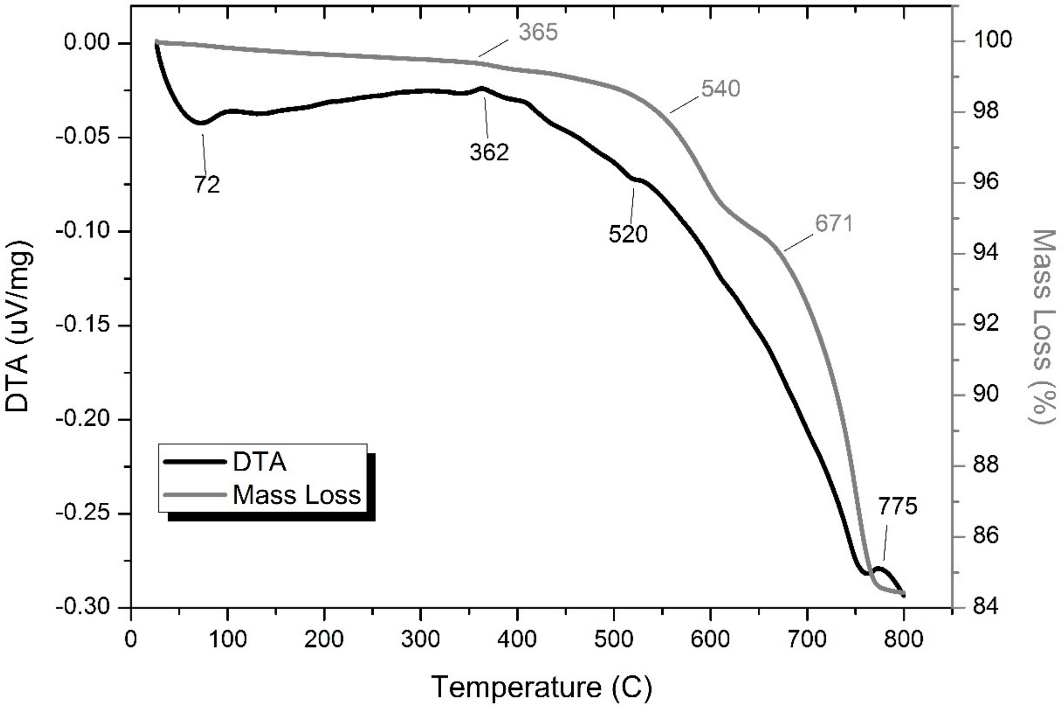

Figure S7. TGA-DTA analysis of "$PdPb_9(VO_4)_6I_2$" subjected to HEBM 30 hr and reacted at 200 °C for 1 hr in a sealed vessel under vacuum.
